# (3*E*,5*E*)-1-Benzyl-3,5-dibenzyl­idenepiperidin-4-one

**DOI:** 10.1107/S1600536809037659

**Published:** 2009-10-17

**Authors:** N. S. Karthikeyan, K. Sathiyanarayanan, P. G. Aravindan, R. S. Rathore

**Affiliations:** a Chemistry Division, School of Science and Humanities, VIT University, Vellore 632 014, India; b Physics Division, School of Science and Humanities, VIT University, Vellore 632 014, India; c Bioinformatics Infrastructure Facility, Department of Biotechnology, School of Life Science, University of Hyderabad, Hyderabad 500 046, India

## Abstract

In the title compound, C_26_H_23_NO, C—H⋯O hydrogen bonds generate a ribbon structure along the *a* axis. These ribbons further assemble into a one-dimensional sheet parallel to the *ac* plane *via* C—H⋯π inter­actions. The piperidin-4-one ring adopts a sofa conformation with the 1-benzyl group in the equatorial position, and the 3- and 5-phenyl substituents stretched out on either side. The benzyl­idene units adopt *E* configurations and the 1-benzyl group is disposed towards the 3- substituent of the piperidin-4-one ring.

## Related literature

For literature related to the synthesis and pharmaceutical activity of 3,5-diaryl­idene-4-piperidone compounds, see Krapcho & Turk (1979 ▶); Sviridenkova *et al.* (2005 ▶); Das *et al.* (2007 ▶). The crystal structures of four analogous compounds have been reported (Suresh *et al.*, 2007 ▶). For ring conformations, see Cremer & Pople (1975 ▶); Duax *et al.* (1976 ▶).
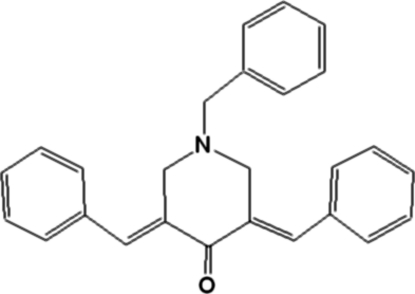


## Experimental

### 

#### Crystal data

C_26_H_23_NO
*M* *_r_* = 365.45
Triclinic, 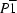

*a* = 6.3354 (4) Å
*b* = 10.2365 (6) Å
*c* = 15.7885 (9) Å
α = 75.245 (2)°
β = 87.651 (3)°
γ = 88.699 (3)°
*V* = 989.24 (10) Å^3^
*Z* = 2
Mo *K*α radiation
μ = 0.07 mm^−1^
*T* = 295 K
0.22 × 0.19 × 0.18 mm

#### Data collection

Bruker APEXII CCD area-detector diffractometer
Absorption correction: multi-scan (*SADABS*; Bruker, 2004 ▶) *T*_min_ = 0.896, *T*_max_ = 0.964
25021 measured reflections
6540 independent reflections
4181 reflections with *I* > 2σ(*I*)
*R*_int_ = 0.035

#### Refinement

*R*[*F* ^2^ > 2σ(*F* ^2^)] = 0.059
*wR*(*F* ^2^) = 0.202
*S* = 1.06
6540 reflections
253 parameters
H-atom parameters constrained
Δρ_max_ = 0.22 e Å^−3^
Δρ_min_ = −0.25 e Å^−3^

### 

Data collection: *APEX2* (Bruker, 2004 ▶); cell refinement: *SAINT-Plus* (Bruker, 2004 ▶); data reduction: *SAINT-Plus*; program(s) used to solve structure: *SHELXS97* (Sheldrick, 2008 ▶); program(s) used to refine structure: *SHELXL97* (Sheldrick, 2008 ▶); molecular graphics: *ORTEP-3* (Farrugia, 1997 ▶); software used to prepare material for publication: *SHELXL97*.

## Supplementary Material

Crystal structure: contains datablocks global, I. DOI: 10.1107/S1600536809037659/bq2159sup1.cif

Structure factors: contains datablocks I. DOI: 10.1107/S1600536809037659/bq2159Isup2.hkl

Additional supplementary materials:  crystallographic information; 3D view; checkCIF report

## Figures and Tables

**Table 1:** Hydrogen-bond geometry (Å, °)

*D*—H⋯*A*	*D*—H	H⋯*A*	*D*⋯*A*	*D*—H⋯*A*
C13—H13⋯N1	0.93	2.57	2.885 (2)	100
C14—H14⋯O1	0.93	2.36	2.7560 (18)	106
C21—H21⋯O1	0.93	2.40	2.761 (2)	103
C2—H2*B*⋯O1^i^	0.97	2.44	3.3798 (17)	163
C16—H16⋯O1^ii^	0.93	2.50	3.304 (2)	145
C7—H7*A*⋯*Cg*2^iii^	0.97	2.77	3.6957 (18)	159
C19—H19⋯*Cg*4^iv^	0.93	2.91	3.523 (2)	125

Symmetry codes: (i) 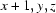; (ii) 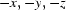; (iii) 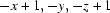; (iv) 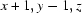. *Cg*2 is the centroid of the C8–C13 ring and *Cg*4 is the centroid of the C22–C27 ring.

**Fig. 1.**
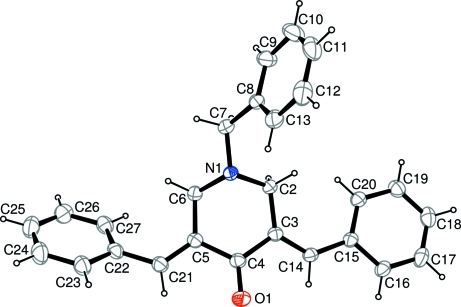
A view of (I) with non-H atoms shown as probability ellipsoids at 30% levels (Farrugia, 2008).

**Fig. 2.**
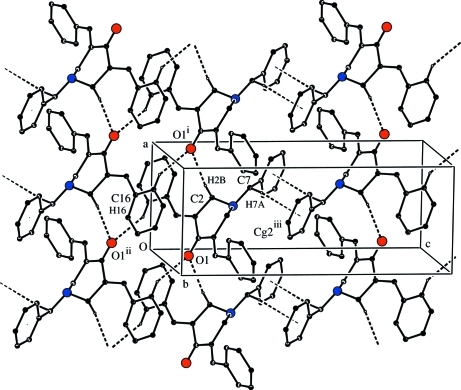
Molecular associations into one-dimensional sheet via C—H···O and C—H···π interactions (see Table 1 for symmetry code). Cg2 is the centroid of (C8—C13) ring.

## supplementary crystallographic information

### Comment

Derivatives of 3,5-diarylidene-4-piperidones (D4P) are pharmaceutically important compounds (Krapcho & Turk, 1979; Sviridenkova *et al.*, 2005; Das *et al.*, 2007). During our investigations on D4P, a series of compounds were prepared. The title compound (3E,5E)-3,5-dibenzylidene-1-phenyl-piperidin-4-one, (I), is reported here.

The molecular structure of (I) with atom numbering scheme is shown in Fig 1. The C3, C5 diene moieties possess E configuration. The C3, C5 phenyl substituents of the piperidinone ring are stretched out on either side with following values of torsion angles: C4—C3—C14—C15 = 176.89 (14)°, C3—C14—C15—C16 = 169.52 (15)°, C4—C5—C21—C22 = -177.30 (14)° and C5—C21—C22—C23 = -138.13 (17)°. The dihedral angle of C3, C5-benzene rings is 41.2 (1)°. The dihedral angles between of benzene rings of C3 and C5-substitutens with respect to the corresponding ring of C1-benzyl substituent are 68.3 (1)° and 69.0 (1)°, respectively.

The *sp*^2^ hybridized C3, C4 and C5 atoms give rise to a sofa conformation of the six-membered piperidinone ring as also observed in the structures of related compounds, namely, (*R*)-3,5-Bis[(*E*)-benzylidene]-1-(1-phenylethyl)piperidin-4-one, 3,5-bis[(*E*)-4-chlorobenzylidene]-1-[(*R*)-1-phenylethyl] piperidin-4-one, and 3,5-bis[(*E*)-2-chlorobenzylidene]-1-[(*R*)-1-phenylethyl] piperidin-4-one (Suresh *et al.*, 2007). In the sofa conformation, the N1 atom is -0.715 (1)Å shifted out of the base plane (C2/C3/C4/C5/C6). The deviation of the ring from ideal sofa conformation, ΔC_2_ (Duax *et al.*, 1976) is 13.3°. The Cremer and Pople (Cremer & Pople, 1975) puckering parameters, corresponding to the ring conformation are as follows: q2 = 0.5420 (15) Å, q3 = 0.2419 (15) Å, φ = 65.95 (14)°, θ = 348.13 (16)°, and the total puckering amplitude Q = 0.5934 (14) Å. The benzyl substituent is in equatorial position of piperidinone ring and its conformation is described by the following torsion angles: C2—N1—C7—C8 = -72.72 (16)°, N1—C7—C8—C9 = 157.64 (14)°. The C1-benzyl group is disposed towards C3-substituent of the piperidin-4-one ring, a feature that varies among related structures.

The observed inter- and intra-molecular interactions are listed in Table 1. The adjacent H14 and H21 atoms participate in an intra-molecular C14—H14···O1···H21—C21 interaction scheme. Additionally, proton H13 of C1-benzyl substituent participate in an intra-molecular C13—H13···N1 interaction.

The crystal packing is characterized by molecular ribbon along *a*-axis due to two C—H···O interactions. They are: C2—H2B···O1 and C16—H16···O1 interactions. These ribbons further assemble *via* C7—H7A···*Cg*2 of an inversion-related molecule leading to a sheet structure parallel to *ac*-plane. *Cg*2 is the centroid of (C8—C13) ring. Crystal packing is shown in Fig2. In addition, the structure also contains a short contact, C19—H19···*Cg*4, where *Cg*4 is the centroid of (C22—C27) ring.

### Experimental

A mixture of 1-benzyl-4-piperidone (0.01 mol) and 2-fluorobenzaldehyde (0.02 mol) was added to a warm solution of ammonium acetate (0.01 mol) in absolute ethanol (15 ml). The mixture was gradually warmed on a water bath until the yellow color changed to orange. The mixture was kept aside overnight at room temperature. Reactions were monitored with TLC for completeness. The solid obtained was separated and the crude compound were purified using silica gel column chromatography with hexane and ethyl acetate as elutant. Final yields: 84.50%; m.p. 427 (2)°K. Suitable single crystals for data collection were grown from ethanol and tetrahydrofurane in (1:1) ratio.

### Refinement

Hydrogen atoms were placed in the geometrically expected positions and refined with the riding options. The distances with hydrogen atoms are: C(aromatic)—H = 0.93 Å, *C*(methylene)—H = 0.97 Å, and *U*_iso_ = 1.2 *U*_eq_(parent)

### Crystal data

**Table:**

C_26_H_23_NO	*Z* = 2
*M**_r_* = 365.45	*F*(000) = 388
Triclinic, *P*1	*D*_x_ = 1.227 Mg m^−^^3^
Hall symbol: -P 1	Melting point: 427 K
*a* = 6.3354 (4) Å	Mo *K*α radiation, λ = 0.71073 Å
*b* = 10.2365 (6) Å	Cell parameters from 1089 reflections
*c* = 15.7885 (9) Å	θ = 2.6–22.0°
α = 75.245 (2)°	µ = 0.07 mm^−^^1^
β = 87.651 (3)°	*T* = 295 K
γ = 88.699 (3)°	Block, yellow
*V* = 989.24 (10) Å^3^	0.22 × 0.19 × 0.18 mm

### Data collection

**Table:**

Bruker APEXII CCD area-detector diffractometer	6540 independent reflections
Radiation source: fine-focus sealed tube	4181 reflections with *I* > 2σ(*I*)
graphite	*R*_int_ = 0.035
φ and ω scans	θ_max_ = 31.6°, θ_min_ = 1.3°
Absorption correction: multi-scan (*SADABS*; Bruker, 2004)	*h* = −9→9
*T*_min_ = 0.896, *T*_max_ = 0.964	*k* = −14→15
25021 measured reflections	*l* = −23→23

### Refinement

**Table:**

Refinement on *F*^2^	Primary atom site location: structure-invariant direct methods
Least-squares matrix: full	Secondary atom site location: difference Fourier map
*R*[*F*^2^ > 2σ(*F*^2^)] = 0.059	Hydrogen site location: inferred from neighbouring sites
*wR*(*F*^2^) = 0.202	H-atom parameters constrained
*S* = 1.06	*w* = 1/[σ^2^(*F*_o_^2^) + (0.1034*P*)^2^ + 0.1348*P*] where *P* = (*F*_o_^2^ + 2*F*_c_^2^)/3
6540 reflections	(Δ/σ)_max_ < 0.001
253 parameters	Δρ_max_ = 0.22 e Å^−^^3^
0 restraints	Δρ_min_ = −0.25 e Å^−^^3^

### Special details

**Table:**

Geometry. All e.s.d.'s (except the e.s.d. in the dihedral angle between two l.s. planes) are estimated using the full covariance matrix. The cell e.s.d.'s are taken into account individually in the estimation of e.s.d.'s in distances, angles and torsion angles; correlations between e.s.d.'s in cell parameters are only used when they are defined by crystal symmetry. An approximate (isotropic) treatment of cell e.s.d.'s is used for estimating e.s.d.'s involving l.s. planes.
Refinement. Refinement of *F*^2^ against ALL reflections. The weighted *R*-factor *wR* and goodness of fit *S* are based on *F*^2^, conventional *R*-factors *R* are based on *F*, with *F* set to zero for negative *F*^2^. The threshold expression of *F*^2^ > σ(*F*^2^) is used only for calculating *R*-factors(gt) *etc*. and is not relevant to the choice of reflections for refinement. *R*-factors based on *F*^2^ are statistically about twice as large as those based on *F*, and *R*- factors based on ALL data will be even larger.

### Fractional atomic coordinates and isotropic or equivalent isotropic displacement parameters (Å^2^)

**Table:**

	*x*	*y*	*z*	*U*_iso_*/*U*_eq_	
N1	0.41574 (19)	0.06361 (11)	0.29573 (7)	0.0388 (3)	
O1	−0.03961 (17)	0.09433 (13)	0.13155 (9)	0.0607 (3)	
C2	0.4799 (2)	0.00037 (14)	0.22535 (9)	0.0388 (3)	
H2A	0.5184	−0.0934	0.2504	0.047*	
H2B	0.6031	0.0458	0.1938	0.047*	
C3	0.3061 (2)	0.00704 (13)	0.16274 (9)	0.0365 (3)	
C4	0.1269 (2)	0.10236 (15)	0.16572 (10)	0.0409 (3)	
C5	0.1623 (2)	0.21277 (14)	0.20919 (9)	0.0391 (3)	
C6	0.3625 (2)	0.20500 (14)	0.25728 (10)	0.0419 (3)	
H6A	0.4758	0.2474	0.2172	0.050*	
H6B	0.3445	0.2525	0.3030	0.050*	
C7	0.5831 (3)	0.05303 (15)	0.35758 (10)	0.0456 (3)	
H7A	0.5509	0.1144	0.3943	0.055*	
H7B	0.7145	0.0818	0.3252	0.055*	
C8	0.6140 (2)	−0.08686 (15)	0.41541 (9)	0.0450 (3)	
C9	0.8058 (3)	−0.1227 (2)	0.45420 (13)	0.0654 (5)	
H9	0.9173	−0.0625	0.4409	0.078*	
C10	0.8331 (4)	−0.2468 (3)	0.51234 (15)	0.0876 (8)	
H10	0.9619	−0.2687	0.5390	0.105*	
C11	0.6736 (5)	−0.3375 (2)	0.53127 (14)	0.0902 (8)	
H11	0.6938	−0.4215	0.5702	0.108*	
C12	0.4837 (4)	−0.3047 (2)	0.49285 (13)	0.0769 (6)	
H12	0.3746	−0.3668	0.5054	0.092*	
C13	0.4529 (3)	−0.17936 (17)	0.43535 (11)	0.0561 (4)	
H13	0.3226	−0.1574	0.4100	0.067*	
C14	0.3014 (2)	−0.06137 (14)	0.10079 (9)	0.0390 (3)	
H14	0.1813	−0.0452	0.0675	0.047*	
C15	0.4524 (2)	−0.15682 (13)	0.07658 (9)	0.0388 (3)	
C16	0.3853 (3)	−0.22865 (16)	0.01835 (10)	0.0474 (3)	
H16	0.2505	−0.2127	−0.0036	0.057*	
C17	0.5152 (3)	−0.32245 (17)	−0.00687 (12)	0.0589 (4)	
H17	0.4671	−0.3696	−0.0453	0.071*	
C18	0.7149 (3)	−0.34718 (17)	0.02409 (12)	0.0604 (5)	
H18	0.8016	−0.4116	0.0075	0.073*	
C19	0.7858 (3)	−0.27558 (18)	0.08004 (12)	0.0547 (4)	
H19	0.9217	−0.2914	0.1009	0.066*	
C20	0.6575 (2)	−0.18052 (15)	0.10546 (10)	0.0467 (3)	
H20	0.7088	−0.1317	0.1423	0.056*	
C21	0.0135 (2)	0.30848 (15)	0.20503 (10)	0.0454 (3)	
H21	−0.1088	0.2984	0.1768	0.055*	
C22	0.0221 (2)	0.42793 (15)	0.24022 (10)	0.0456 (3)	
C23	−0.1555 (3)	0.46678 (18)	0.28275 (12)	0.0582 (4)	
H23	−0.2784	0.4167	0.2888	0.070*	
C24	−0.1519 (4)	0.5785 (2)	0.31605 (14)	0.0711 (6)	
H24	−0.2713	0.6023	0.3453	0.085*	
C25	0.0267 (4)	0.6549 (2)	0.30642 (14)	0.0715 (6)	
H25	0.0285	0.7303	0.3290	0.086*	
C26	0.2030 (3)	0.61958 (17)	0.26319 (13)	0.0631 (5)	
H26	0.3238	0.6719	0.2559	0.076*	
C27	0.2011 (3)	0.50666 (15)	0.23063 (11)	0.0520 (4)	
H27	0.3215	0.4830	0.2019	0.062*	

### Atomic displacement parameters (Å^2^)

**Table:**

	*U*^11^	*U*^22^	*U*^33^	*U*^12^	*U*^13^	*U*^23^
N1	0.0467 (7)	0.0396 (6)	0.0315 (6)	0.0051 (5)	−0.0077 (5)	−0.0109 (4)
O1	0.0388 (6)	0.0723 (8)	0.0803 (9)	0.0045 (5)	−0.0156 (6)	−0.0349 (7)
C2	0.0413 (7)	0.0429 (7)	0.0344 (7)	0.0036 (5)	−0.0053 (5)	−0.0138 (5)
C3	0.0367 (7)	0.0374 (6)	0.0342 (7)	−0.0019 (5)	−0.0018 (5)	−0.0071 (5)
C4	0.0345 (7)	0.0462 (7)	0.0420 (8)	−0.0016 (5)	−0.0016 (6)	−0.0112 (6)
C5	0.0396 (7)	0.0415 (7)	0.0350 (7)	0.0005 (5)	−0.0003 (5)	−0.0078 (5)
C6	0.0482 (8)	0.0384 (6)	0.0401 (7)	0.0028 (5)	−0.0077 (6)	−0.0108 (5)
C7	0.0523 (9)	0.0478 (7)	0.0398 (8)	0.0025 (6)	−0.0117 (6)	−0.0156 (6)
C8	0.0563 (9)	0.0497 (8)	0.0322 (7)	0.0127 (6)	−0.0089 (6)	−0.0162 (6)
C9	0.0654 (11)	0.0815 (12)	0.0521 (10)	0.0227 (9)	−0.0187 (8)	−0.0216 (9)
C10	0.1057 (19)	0.0971 (17)	0.0605 (13)	0.0531 (15)	−0.0312 (12)	−0.0211 (12)
C11	0.161 (3)	0.0623 (12)	0.0441 (10)	0.0447 (15)	−0.0169 (14)	−0.0096 (9)
C12	0.1278 (19)	0.0521 (10)	0.0483 (10)	0.0019 (11)	0.0043 (11)	−0.0094 (8)
C13	0.0736 (11)	0.0530 (9)	0.0420 (9)	0.0042 (8)	−0.0071 (8)	−0.0123 (7)
C14	0.0404 (7)	0.0416 (6)	0.0351 (7)	−0.0015 (5)	−0.0064 (5)	−0.0090 (5)
C15	0.0473 (8)	0.0387 (6)	0.0296 (6)	−0.0019 (5)	−0.0020 (5)	−0.0070 (5)
C16	0.0579 (9)	0.0483 (8)	0.0381 (8)	−0.0040 (6)	−0.0061 (6)	−0.0139 (6)
C17	0.0847 (13)	0.0495 (8)	0.0488 (9)	−0.0041 (8)	−0.0005 (9)	−0.0240 (7)
C18	0.0781 (13)	0.0487 (8)	0.0556 (10)	0.0084 (8)	0.0069 (9)	−0.0176 (7)
C19	0.0548 (10)	0.0577 (9)	0.0512 (9)	0.0107 (7)	−0.0018 (7)	−0.0141 (7)
C20	0.0489 (9)	0.0516 (8)	0.0422 (8)	0.0026 (6)	−0.0053 (6)	−0.0162 (6)
C21	0.0415 (8)	0.0483 (7)	0.0461 (8)	0.0039 (6)	−0.0028 (6)	−0.0117 (6)
C22	0.0500 (8)	0.0416 (7)	0.0418 (8)	0.0091 (6)	−0.0039 (6)	−0.0048 (6)
C23	0.0563 (10)	0.0592 (9)	0.0601 (11)	0.0085 (7)	0.0023 (8)	−0.0186 (8)
C24	0.0826 (14)	0.0709 (12)	0.0630 (12)	0.0188 (10)	0.0070 (10)	−0.0261 (10)
C25	0.1052 (17)	0.0520 (9)	0.0608 (12)	0.0106 (10)	−0.0095 (11)	−0.0208 (8)
C26	0.0816 (13)	0.0429 (8)	0.0612 (11)	−0.0042 (8)	−0.0094 (9)	−0.0049 (7)
C27	0.0568 (10)	0.0423 (7)	0.0519 (9)	0.0035 (6)	0.0001 (7)	−0.0033 (6)

### Geometric parameters (Å, °)

**Table:**

N1—C7	1.4543 (18)	C13—H13	0.9300
N1—C6	1.4575 (18)	C14—C15	1.4610 (19)
N1—C2	1.4617 (17)	C14—H14	0.9300
O1—C4	1.2183 (17)	C15—C20	1.390 (2)
C2—C3	1.4984 (19)	C15—C16	1.399 (2)
C2—H2A	0.9700	C16—C17	1.375 (2)
C2—H2B	0.9700	C16—H16	0.9300
C3—C14	1.3417 (19)	C17—C18	1.370 (3)
C3—C4	1.4870 (19)	C17—H17	0.9300
C4—C5	1.489 (2)	C18—C19	1.377 (3)
C5—C21	1.3354 (19)	C18—H18	0.9300
C5—C6	1.495 (2)	C19—C20	1.380 (2)
C6—H6A	0.9700	C19—H19	0.9300
C6—H6B	0.9700	C20—H20	0.9300
C7—C8	1.503 (2)	C21—C22	1.469 (2)
C7—H7A	0.9700	C21—H21	0.9300
C7—H7B	0.9700	C22—C27	1.387 (2)
C8—C13	1.380 (3)	C22—C23	1.389 (2)
C8—C9	1.383 (2)	C23—C24	1.376 (3)
C9—C10	1.376 (3)	C23—H23	0.9300
C9—H9	0.9300	C24—C25	1.371 (3)
C10—C11	1.360 (4)	C24—H24	0.9300
C10—H10	0.9300	C25—C26	1.374 (3)
C11—C12	1.366 (4)	C25—H25	0.9300
C11—H11	0.9300	C26—C27	1.380 (2)
C12—C13	1.385 (3)	C26—H26	0.9300
C12—H12	0.9300	C27—H27	0.9300
			
C7—N1—C6	110.28 (11)	C8—C13—C12	120.50 (19)
C7—N1—C2	111.05 (11)	C8—C13—H13	119.8
C6—N1—C2	108.77 (11)	C12—C13—H13	119.8
N1—C2—C3	111.46 (11)	C3—C14—C15	131.10 (13)
N1—C2—H2A	109.3	C3—C14—H14	114.4
C3—C2—H2A	109.3	C15—C14—H14	114.4
N1—C2—H2B	109.3	C20—C15—C16	117.51 (13)
C3—C2—H2B	109.3	C20—C15—C14	125.51 (13)
H2A—C2—H2B	108.0	C16—C15—C14	116.97 (13)
C14—C3—C4	116.30 (12)	C17—C16—C15	121.06 (16)
C14—C3—C2	125.73 (12)	C17—C16—H16	119.5
C4—C3—C2	117.90 (11)	C15—C16—H16	119.5
O1—C4—C3	121.95 (13)	C18—C17—C16	120.63 (16)
O1—C4—C5	120.88 (13)	C18—C17—H17	119.7
C3—C4—C5	117.12 (12)	C16—C17—H17	119.7
C21—C5—C4	118.78 (13)	C17—C18—C19	119.27 (15)
C21—C5—C6	124.66 (13)	C17—C18—H18	120.4
C4—C5—C6	116.54 (11)	C19—C18—H18	120.4
N1—C6—C5	109.06 (11)	C18—C19—C20	120.70 (17)
N1—C6—H6A	109.9	C18—C19—H19	119.7
C5—C6—H6A	109.9	C20—C19—H19	119.7
N1—C6—H6B	109.9	C19—C20—C15	120.77 (15)
C5—C6—H6B	109.9	C19—C20—H20	119.6
H6A—C6—H6B	108.3	C15—C20—H20	119.6
N1—C7—C8	113.90 (12)	C5—C21—C22	127.05 (14)
N1—C7—H7A	108.8	C5—C21—H21	116.5
C8—C7—H7A	108.8	C22—C21—H21	116.5
N1—C7—H7B	108.8	C27—C22—C23	118.09 (16)
C8—C7—H7B	108.8	C27—C22—C21	122.26 (14)
H7A—C7—H7B	107.7	C23—C22—C21	119.63 (15)
C13—C8—C9	118.31 (16)	C24—C23—C22	120.79 (18)
C13—C8—C7	122.09 (14)	C24—C23—H23	119.6
C9—C8—C7	119.49 (16)	C22—C23—H23	119.6
C10—C9—C8	120.6 (2)	C25—C24—C23	120.40 (18)
C10—C9—H9	119.7	C25—C24—H24	119.8
C8—C9—H9	119.7	C23—C24—H24	119.8
C11—C10—C9	120.7 (2)	C24—C25—C26	119.73 (18)
C11—C10—H10	119.7	C24—C25—H25	120.1
C9—C10—H10	119.7	C26—C25—H25	120.1
C10—C11—C12	119.7 (2)	C25—C26—C27	120.11 (19)
C10—C11—H11	120.2	C25—C26—H26	119.9
C12—C11—H11	120.2	C27—C26—H26	119.9
C11—C12—C13	120.3 (2)	C26—C27—C22	120.86 (16)
C11—C12—H12	119.9	C26—C27—H27	119.6
C13—C12—H12	119.9	C22—C27—H27	119.6
			
C7—N1—C2—C3	178.28 (12)	C7—C8—C13—C12	−176.30 (15)
C6—N1—C2—C3	−60.18 (15)	C11—C12—C13—C8	0.8 (3)
N1—C2—C3—C14	−167.71 (13)	C4—C3—C14—C15	176.89 (14)
N1—C2—C3—C4	15.50 (17)	C2—C3—C14—C15	0.1 (2)
C14—C3—C4—O1	18.9 (2)	C3—C14—C15—C20	−11.5 (3)
C2—C3—C4—O1	−164.04 (14)	C3—C14—C15—C16	169.52 (15)
C14—C3—C4—C5	−158.55 (13)	C20—C15—C16—C17	2.2 (2)
C2—C3—C4—C5	18.54 (18)	C14—C15—C16—C17	−178.79 (14)
O1—C4—C5—C21	−4.5 (2)	C15—C16—C17—C18	−0.5 (3)
C3—C4—C5—C21	173.00 (13)	C16—C17—C18—C19	−0.9 (3)
O1—C4—C5—C6	173.68 (14)	C17—C18—C19—C20	0.5 (3)
C3—C4—C5—C6	−8.87 (19)	C18—C19—C20—C15	1.3 (3)
C7—N1—C6—C5	−168.16 (12)	C16—C15—C20—C19	−2.6 (2)
C2—N1—C6—C5	69.84 (14)	C14—C15—C20—C19	178.48 (15)
C21—C5—C6—N1	144.04 (14)	C4—C5—C21—C22	−177.30 (14)
C4—C5—C6—N1	−33.97 (17)	C6—C5—C21—C22	4.7 (3)
C6—N1—C7—C8	166.64 (12)	C5—C21—C22—C27	43.6 (2)
C2—N1—C7—C8	−72.72 (16)	C5—C21—C22—C23	−138.13 (17)
N1—C7—C8—C13	−26.1 (2)	C27—C22—C23—C24	−1.4 (3)
N1—C7—C8—C9	157.64 (14)	C21—C22—C23—C24	−179.78 (17)
C13—C8—C9—C10	−1.1 (3)	C22—C23—C24—C25	1.1 (3)
C7—C8—C9—C10	175.31 (17)	C23—C24—C25—C26	0.0 (3)
C8—C9—C10—C11	1.4 (3)	C24—C25—C26—C27	−0.9 (3)
C9—C10—C11—C12	−0.7 (3)	C25—C26—C27—C22	0.5 (3)
C10—C11—C12—C13	−0.4 (3)	C23—C22—C27—C26	0.6 (2)
C9—C8—C13—C12	0.0 (2)	C21—C22—C27—C26	178.91 (15)

### Hydrogen-bond geometry (Å, °)

**Table:**

*D*—H···*A*	*D*—H	H···*A*	*D*···*A*	*D*—H···*A*
C13—H13···N1	0.93	2.57	2.885 (2)	100
C14—H14···O1	0.93	2.36	2.7560 (18)	106
C21—H21···O1	0.93	2.40	2.761 (2)	103
C2—H2B···O1^i^	0.97	2.44	3.3798 (17)	163
C16—H16···O1^ii^	0.93	2.50	3.304 (2)	145
C7—H7A···Cg2^iii^	0.97	2.77	3.6957 (18)	159
C19—H19···Cg4^iv^	0.93	2.91	3.523 (2)	125

Symmetry codes: (i) *x*+1, *y*, *z*; (ii) −*x*, −*y*, −*z*; (iii) −*x*+1, −*y*, −*z*+1; (iv) *x*+1, *y*−1, *z*.
